# SELF CARE TRAININGS FOR NCD MANAGEMENT IN OLD AGE IN INDIA

**DOI:** 10.1093/geroni/igad104.0009

**Published:** 2023-12-21

**Authors:** Prakash Tyagi

**Affiliations:** GRAVIS, Jodphur, Rajasthan, India

## Abstract

With over 100 million older people, India has the second largest elderly population in the world. Older people in many parts of India, especially in the rural settings live in vulnerable conditions with limited facilities and with an overall poor health status. GRAVIS has been working actively in the Thar Desert of India to address older people’s health. It has multi-layered health intervention combining medical services, public health activities and research component. Self-care training is integral part of our work with older people. With help of locally designed contents, GRAVIS team trains older people on healthy ageing. The curriculum includes 10 modules focusing on basics of human body and biology of ageing, Common diseases in old age, prevention of common diseases, management of common diseases and useful information on Government health programmes. Older people in groups of 12-15 attend trainings once in 15 days. To provide service to older people and to take self care education further, GRAVIS also works with a cadre of Village based Health Workers (VHWs). The impact of our self-care training interventions has been quite positive. The trainings are well received within the community. Over 1,500 older people have been trained and over 85% report a significant increase in their knowledge levels. GRAVIS has been able to draw the attention of local health authorities and hopes that the trainings could be scaled up further. Self care and improved health knowledge are critically important for older people to stay healthy, particularly in remote and resource scarce settings.

